# Effectiveness of an Occupational Well-being Intervention Among Nurse Educators: A Quasi-Experimental Study

**DOI:** 10.1097/NNE.0000000000001482

**Published:** 2023-08-03

**Authors:** Jenni Rinne, Helena Leino-Kilpi, Terhi Saaranen, Mika P. Tarvainen, Miko Pasanen, Sanna Koskinen, Leena Salminen

**Affiliations:** Doctoral researcher (Ms Rinne), Professor (Drs Leino-Kilpi and Salminen), Statistician (Dr Pasanen), and Postdoctoral Researcher (Dr Koskinen), Department of Nursing Science, University of Turku, Turku, Finland; Nurse Director (Drs Leino-Kilpi and Salminen), Turku University Hospital, Turku, Finland; Professor (Dr Saaranen), Department of Nursing Science, University of Eastern Finland, Kuopio, Finland; Senior Researcher (Dr Tarvainen), Department of Technical Physics, University of Eastern Finland, Kuopio, Finland; and ... (Dr Tarvainen), Department of Clinical Physiology and Nuclear Medicine, Kuopio University Hospital, Kuopio, Finland.

**Keywords:** digital health intervention, heart rate variability, nurse educator, occupational well-being, recovery experiences

## Abstract

**Background:**

Occupational well-being supports the retention of the nurse educator workforce and their ability to manage workload. There is a research gap regarding interventions promoting occupational well-being.

**Purpose:**

To evaluate the effectiveness of an 8-workweek digital occupational well-being intervention using self-conducted exercises among nurse educators in secondary vocational nursing schools in Finland.

**Methods:**

A quasi-experimental study design was applied using an intervention group (n = 37) and a control group (n = 40). Data were collected at baseline, post, and 1-month follow-up using a questionnaire and a heart rate sensor to assess the resource-workload-balance and its associating and promoting factors.

**Results:**

This study found no statistical effects on the resource-workload-balance. Positive effects were found on associating factors (general well-being) and promoting factors (recovery experiences and self-regulation).

**Conclusions:**

Self-Help INtervention for Educators (SHINE) has the potential to promote recovery experiences during working hours; however, the intervention needs more investigation.

There is a shortage of nurse educators due to retirement and attrition in the profession,^1^ and educators are experiencing heavy workload affecting their occupational well-being (OW).^2^–^4^ The global challenge of shortage in the nursing workforce has also set demands on investing in nursing education.^4^,^5^ OW is at an inadequate level because of high workload, the lack of support for personal resources, and the inability to take breaks.^2^,^3^ Nurse educators deserve support for their personal resources at work, and this requires more evidence-based interventions.^6^,^7^

## Background

The study applied the Content Model for the Promotion of School Community Staff's Occupational Well-being^8^ as a theoretical framework. In this model, the overall OW of educators is defined by 4 aspects of working life: (1) workers' resources and work (eg, person's health, fitness, vigor, and workload); (2) work community (eg, collegiality); (3) working conditions (eg, physical factors); and (4) professional competence (eg, pedagogical skills).^2^,^8^ The focus of this study is in the first of these 4 aspects, the personal resource-workload-balance.

The resource-workload-balance consists of personal resources at work, workload factors, and the balance between them. Personal resources at work are an individual's perspective regarding their own physical and mental health, fitness, and vigor. They involve the individual's abilities to perform daily activities with endurance, strength, flexibility, and energy at work (eg, physical energy, self-efficacy, resilience, and cognitive liveliness).^2^,^8^ Personal workload factors are the physical and mental stress experienced by educators in their work. The workload, and especially the mental workload, has been found to be too heavy and consequently needing better workload management.^2^,^3^ In addition to self-reported experiences of the personal resources and workload, the physical state of the autonomic nervous system can provide objective information about the resource-workload-balance. Heart rate variability (HRV), which measures the normal variation in the time intervals between consecutive heart beats (R-R intervals), is a commonly used tool to assess autonomic nervous system activity.^9^ A high resting HRV is an indication of good recovery and a high ability to tolerate stress, that is, high resource-workload-balance.^10^

Self-Help INtervention for Educators (SHINE) was created to be applicable to the daily working life of educators working in nursing education needing better resource-workload-balance.^2^,^3^ There are 4 resource-workload-balance promoting components implemented in SHINE: (1) physical activity at work; (2) recovery activities at work; (3) self-regulation development activities at work; and (4) workplace support for personal resources promoting activities at work. The aim of SHINE is to promote the resource-workload-balance (primary outcome); its associated factors (overall OW and general well-being [GW], secondary outcomes); and its promotive factors (physical activity, recovery experiences, self-regulation, and workplace support, condition outcomes) (see Supplemental Digital Content, Figure 1, available at: http://links.lww.com/NE/B363).

Overall OW and GW are closely connected to the resource-workload-balance being associating factors in this study. Overall OW is described as the best possible level of affective feelings toward work,^8^ whereas GW encompasses subjective feelings of satisfaction and happiness in everyday life.^11^,^12^ It has been shown that individuals with a good resource-workload-balance have a better experience regarding overall OW^2^ and also better GW.^11^

The 4 components of the intervention are those promotive factors that have beneficial outcomes in promoting the physical or mental resources of educators.^6^ Physical activity means less sitting and more engagement in moderate-intensity activity associated with well-being benefits^13^; this study was interested in occupational physical activity and sitting time.^14^ Physical activity at work is beneficial for the resources of workers (eg, lower anxiety and higher HRV).^15^,^16^ Nurse educators face health problems due to long working hours and prolonged sitting time, needing extra physical activity.^17^ Recovery during working hours is possible when engaging in activities promoting psychological detachment and relaxation where psychophysiological functioning returns to a level where there are no stressors.^18^ In previous studies, relaxation during breaks, exposure to nature, and short detachment exercises have been shown to generate recovery.^19^,^20^ Self-regulation is important when promoting health behavior changes,^21^ which has also been found to affect the way individuals use personal resources during stressful work situations.^22^ Activities such as awareness, self-reflection, and self-monitoring have been found to develop self-regulation of personal resources at work.^21^ Self-reflection has beneficial OW effects on nurse educators reflecting gratitude at work^23^ and alleviating work strain when understanding and being aware of the psychological processes.^7^

Workplace support is the support from managers and work colleagues for activities to promote personal resources, which is highly valued but inadequate among educators working in nursing education.^2^,^3^ Moreover, workplace support and the involvement of managers in an intervention development and delivery are likely to promote the adoption of well-being actions at work.^6^ The study hypothesis was that SHINE will improve nurse educators' resource-workload-balance and the associated and promoting factors compared with their level prior to the intervention and when compared with a control group (CG) both in post and 1-month follow-up periods.

## Methods

### Design and Sample

This controlled quasi-experimental study used 3 measurement points to evaluate the intervention's effectiveness: baseline (*T*_0_), post (*T*_1_), and 1-month follow-up (*T*_2_) (see Supplemental Digital Content, Figure 2, available at: http://links.lww.com/NE/B364).^24^ This study was granted ethical approval by the Ethics Committee for Human Sciences at the University of Turku, Health Care Division (November 2021), and the study protocol is registered in the ClinicalTrials.gov database (NCT05307107).^25^

Participants were recruited from vocational secondary nursing schools educating practical nurses (corresponding to licensed practical nurses) in the southern part of Finland. The participating schools were similar (eg, management and study programs) and were randomly assigned in order of enrollment to either the intervention group (IG, n = 2) or the CG (n = 3) until a sufficient sample size was reached. The sample size was confirmed by calculating the power analysis with an established α level of .05 and the power of 0.80 for the primary outcomes (self-reported resource-workload-balance). This calculation was made after the questionnaire's pilot study (n = 26) indicated that a group of 37 was needed to show a 0.5-point difference within the IG and the CG with an effect size of 0.6. Managers organized informative Microsoft Teams session where the research team informed and recruited voluntary educators to participate in the study. The enrolled educators (n = 80) had full-time work contracts as professional specialized educators working in educational settings. The educators were specialized teaching nursing, social care, rehabilitation, or general subjects (eg, languages or mathematics), as part of the national regulated practical nurse program.^26^

The educators received oral and written information and gave their informed consent. The excluding criteria were pregnancy and having a pacemaker because of the HRV measurements. There were 3 dropouts for personal reasons during the study (see Supplemental Digital Content, Figure 2, available at: http://links.lww.com/NE/B364), with a final sample of 77. The demographic background of educators was similar; however, statistical calculations on the correspondence of these 2 groups might produce speculative results and be at risk of misinterpretation.^27^ When comparing groups, for example, the mean age was 49.05 ± 8.09 years in the IG and 50.40 ± 7.92 years in the CG and the mean work experience in years was 11.05 ± 6.98 and 11.78 ± 8.21 for the IG and the CG, respectively (see Supplemental Digital Content, Table 1, available at: http://links.lww.com/NE/B365).

### Intervention

SHINE is a self-conductive intervention in the daily working life of educators working in nursing education wherever their work takes place. This intervention was piloted before the intervention enrollment with a 4-week one-group design (n = 9). The pilot included development of the program to make it more acceptable in the working life of educators.

SHINE is constructed from 4 key components and was delivered as a digital web-based Smart Break SHINE program with support arranged in the workplace (see Supplemental Digital Content, Figure 3, available at: http://links.lww.com/NE/B366). The program was modified from the original Smart Break program (https://www.smart-break.com) by adding a SHINE module especially developed for this study; this module required weekly self-regulative task and breathing exercises in addition to the existing physical exercises. Educators logged into the program with their computer or mobile device. The program instructed the educators to do the 3-minute break exercises twice during the working day and provided weekly changing self-regulative tasks (5-10 minutes per workday); these had different options suitable for everyone to complete independently or with colleagues. In addition, the program guided educators to self-reflect on the level of their personal resources once a week. The program stored information on the activities completed to capture the fidelity of the intervention.

Written instructions for the program were given to each participant, and voluntary Microsoft Teams sessions were offered to assist with the intervention procedures. Email reminders were sent to the participants by the research team during each starting week of the intervention to reiterate the need to do the program's exercises. In addition, remainders could be set in the program to provide a maximum of 3 reminders per day. Previous research indicates that positive health-related results for individuals can be achieved with interventions lasting 1 to 3 months^6^; therefore, an 8-week intervention was chosen.

### Instruments and Data Collection

An electronic self-report questionnaire and HRV measurements with a heart rate sensor were administered at 3 different time points (*T*_0_-*T*_2_) (see Supplemental Digital Content, Table 2, available at: http://links.lww.com/NE/B367). The questionnaire consisted of a total of 45 items, of which 8 were demographic items (gender not reported), 17 were developed for this study, and 20 items were from other instruments. The developed items were examined by an expert panel (n = 6) and were subject to a questionnaire piloting (n = 26) the content. After the piloting, the following factors were calculated: the content validity index of the scale (CVI-S)^28^ of the question's relevance (r) and the understandability (u) before the research. The Cronbach α for the questionnaire's subscales was calculated (if applicable) from the questionnaire piloting data (n = 26) and the baseline data of this study (n = 80).^29^ Permissions to use the existing items were obtained from the copyright holders.

In the data collection process, the questionnaire was sent as a personal link to the educator's email from REDCap (Research Electronic Data Capture). Reminder emails (n = 2) were sent to nonresponding educators at each time point. In addition to the questionnaire, educators were asked to take short 3-minute resting HRV measurements every workday morning; the measurements were taken soon after wake-up during the measurement weeks using a Kubios HRV mobile application (Kubios Oy, Kuopio, Finland),^30^ connected to a Polar H10 or H7 heart rate sensor (Polar Electro Oy, Kempele, Finland). At least 2 good-quality measurements for each measurement week/participant were required to continue with further analysis.

### Data Analysis

The HRV data were preprocessed (signal quality assessment and beat correction) and analyzed with Kubios HRV Premium (ver.3.5) software (Kubios Oy). Three HRV parameters were chosen for analyses: (1) mean R-R interval (reflecting resting heart rate); (2) root mean square of successive R-R interval differences (RMSSD, reflecting parasympathetic nervous activity); and (3) ratio between Poincaré plot standard deviations SD_1_/SD_2_ (reflecting balance between parasympathetic and sympathetic branches).^9^,^10^ At each measurement time point (*T*_0_-*T*_2_), the mean of the 2 or more weekly HRV parameter values of each individual was reported.

The data were analyzed using 2 statistical programs, SPSS v27 (IBM Corp, Armonk, New York) and R version 4.0.2. The descriptive statistics are represented as frequencies, percentages, and mean and SD values. The use of a linear mixed model (LMM) was applicable to assess the changes at several time points (*T*_0_-*T*_2_) presenting estimated coefficients with confidence interval (95% CI).^31^ The *P* value was calculated setting the significance at .05. Within-group effect sizes (LMM's equivalent Cohen's *d* with 95% CI) were calculated using an R package emmeans version 1.6.1, and between-group effect sizes were calculated with emmeans version 1.8.6 according to LMM values.^29^

## Results

There was high engagement by the participants in the intervention. Of the participating educators, 81% completed 6 to 8 of the 8 weekly self-regulative tasks during the 8 workweeks. In addition, 70% of the educators completed both daily 3-minute exercises 3 to 5 times per workweek. There were only 2 dropouts in the intervention.

### Primary Outcomes

The self-reported resource-workload-balance was considered moderately good in both groups at baseline (Table). In the comparison between the groups, no significant effects were found for the SHINE intervention on the resource-workload-balance, RSW and HRV (see Supplemental Digital Content, Table 3, available at: http://links.lww.com/NE/B368). In the CG, the resting heart rate had statistically increased (*P* < .05, *d* = −0.66; 95% CI, −1.182 to −0.1414) in the 1-month follow-up period, indicating a worsening of the HRV results (see Supplemental Digital Content, Table 3, available at: http://links.lww.com/NE/B368).

**Table.** Change of Variables in the IG and the CG^a^

Variables (Scale)	Baseline (T_0_)		Post (T_1_)		1-mo Follow-up (T_2_)	
	IG (n = 30)	CG (n = 31)	IG (n = 30)	CG (n = 31)	IG (n = 29)	CG (n = 29)
*Primary outcomes*						
HRV						
Mean R-R, ms	945.09 ± 100.99	968.63 ± 125.08	923.08 ± 83.47	957.64 ± 96.07	933.38 ± 86.44	946.05 ± 103.08
RMSSD, ms	28.79 ± 17.67	36.75 ± 28.15	26.26 ± 13.66	33.97 ± 17.86	29.39 ± 14.11	33.78 ± 17.46
SD_2_/SD_1_ (range)	2.35 ± 0.59	2.03 ± 0.45	2.30 ± 0.59	1.97 ± 0.42	2.23 ± 0.57	2.01 ± 0.50
	**IG (n = 37)**	**CG (n = 40)**	**IG (n = 37)**	**CG (n = 40)**	**IG (n = 37)**	**CG (n = 40)**
RWB (1-5)	3.45 ± 0.65	3.44 ± 0.57	3.46 ± 0.64	3.45 ± 0.63	3.47 ± 0.69	3.40 ± 0.67
Resources	3.75 ± 0.59	3.66 ± 0.69	3.71 ± 0.6	3.64 ± 0.68	3.69 ± 0.59	3.55 ± 0.66
Workload	3.19 ± 0.72	3.24 ± 0.64	3.21 ± 0.68	3.28 ± 0.67	3.25 ± 0.79	3.26 ± 0.71
Balance	3.41 ± 0.76	3.42 ± 0.66	3.46 ± 0.74	3.44 ± 0.76	3.46 ± 0.78	3.40 ± 0.78
*Secondary outcomes*						
Overall OW (0-5)	3.22 ± 1.02	3.15 ± 1.15	3.37 ± 0.98	3.16 ± 1.12	3.39 ± 1.05	3.03 ± 1.23
GW (0-5)	3.90 ± 0.8	3.82 ± 0.77	3.99 ± 0.89	3.74 ± 0.91	4.17 ± 0.76	3.74 ± 0.85
*Condition outcomes*						
PA (sitting %)	55.22 ± 22.94	54.55 ± 22.99	54.46 ± 25.02	45.62 ± 24.44	53.51 ± 25.00	48.30 ± 22.44
RE (1-5)	2.34 ± 0.96	2.16 ± 0.82	2.94 ± 1.00	2.27 ± 0.85	2.82 ± 0.99	2.19 ± 0.83
SR (1-5)	3.39 ± 0.51	3.33 ± 0.47	3.48 ± 0.56	3.45 ± 0.48	3.57 ± 0.58	3.39 ± 0.58
WS (1-5)	3.15 ± 0.84	3.23 ± 0.79	3.29 ± 0.86	3.36 ± 0.79	3.31 ± 0.88	3.23 ± 0.73

Abbreviations: CG, control group; GW, general well-being; HRV, heart rate variability; IG, intervention group; OW, occupational well-being; PA, physical activity; RE, recovery; RMSSD, root mean square of successive R-R interval differences; RWB, resource-workload-balance; SR, self-regulation; WS, workplace support.

^a^The values given are mean ± SD.

### Secondary Outcomes

Overall OW and GW were considered moderately good in both groups at baseline (Table). SHINE was seen as effective in promoting GW within the IG (*P* < .05, *d* = 0.63; 95% CI, 0.1646 to 1.091) and comparing the groups at the 1-month follow-up period (*P* < .05, *d* = −0.79; 95% CI, −1.44 to −0.150). The overall OW did not demonstrate statistically significant effects (see Supplemental Digital Content, Table 3, available at: http://links.lww.com/NE/B368).

### Condition Outcomes

Perceived weekly sitting time at work was more than 50%, recovery experiences were moderately low, and self-regulation of personal resources and workplace support were moderately good in both groups at baseline (Table). The IG found no significant effect regarding the promotion of physical activity at work. Within the group comparisons, the CG had a statistically significant decrease in perceived sitting time in the postmeasurement (*P* < .05, *d* = −0.64; 95% CI, −1.086 to −0.19433) but indicated no gained beneficial results in the 1-month follow-up period. SHINE was statistically effective in promoting recovery experiences at work both in *T*_1_ and *T*_2_ measurements within the IG (*T*_1_: *P* < .001, *d* = 1.16; 95% CI, 0.683 to 1.627; *T*_2_: *P* < .001, *d* = 0.92; 95% CI, 0.454 to 1.389) and between-group comparison (*T*_1_: *P* < .01, *d* = −0.95; 95% CI, −1.598 to −0.304; *T*_2_: *P* < .01, *d* = −0.86; 95% CI, −1.506 to −0.217). In the 1-month follow-up period (*T*_2_), the self-regulation of personal resources in the IG statistically increased compared with the baseline (*P* < .05, *d* = 0.59; 95% CI, 0.1250 to 1.050) (see Supplemental Digital Content, Table 3, available at: http://links.lww.com/NE/B368). No SHINE effects were found in workplace support for activities promoting personal resources.

## Discussion

This study hypothesis was that the personal resource-workload-balance of nurse educators would significantly increase; however, no significant effects were found. A previous study found better HRV after 3 months of the intervention including daily walking^16^; however, this study found no such effects. However, HRV values were significantly worse in the CG between baseline and 1-month follow-up, although the weekly working hours and sitting time at work significantly decreased. Concurrently, the working hours in the IG remained the same and no significant changes were found in HRV values. This could indicate that there are positive associations of the intervention with sustaining the long-term resource-workload-balance.

This study partly supported the stated hypothesis for the secondary outcomes. GW showed statistical effects for SHINE in the 1-month follow-up period. The intervention components were primarily aimed at promoting the educators' resources at work; however, the direct benefits were observed in GW, promoting affective feelings in daily life. A previous study showed direct associations between OW and GW^11^; the findings of this study showed near significant effects for increased overall OW in the 1-month follow-up period (*P* = .055, *d* = −0.62; 95% CI, −1.265 to 0.0175).

Considering the condition outcomes, moderately good results were demonstrated by the significant increase in recovery experiences and self-regulation of personal resources at work. This study supports the evidence that recovery experiences at work increase when recovery activities are encouraged in daily working life.^19^,^20^ This observation adds important evidence to the need to protect workers from the possible negative consequences of a poor resource-workload-balance. The ratings for the self-regulation of personal resources at work were statistically better within the IG at the 1-month follow-up period, indicating a better development of awareness, goal setting, and self-monitoring. These factors were found to be important in maintaining health changes^21^ and preventing exhaustion; they were also found to need management support.^22^

Finally, a few comments about the intervention's fidelity and content. The participants' responsiveness was generally good, the dropout rate was low, and there was active engagement by the participants in the IG. However, further research is required on the acceptability of the exercises and development needs. The IG needed motivation and determination to complete all the activities included. Only a few studies were found showing the possibilities of using self-conductive exercises related to the expression of gratitude at work among nurse educators.^23^ Future studies should include an intervention delivery with self-conducted assessable exercises preferably with a longer follow-up period to discover the longitudinal effects and any necessary support. Self-conducted exercises can partly resolve the cost-effectiveness issues, as these exercises do not need constant facilitators.

### Strengths and Limitations

There are strengths in regard to the setting and content of the intervention. This intervention was designed to fit into the working life of educators working in nursing education using a guided program. The program consisted of evidence-based exercises that aimed at becoming habitual during the 8 workweeks of the intervention. These exercises use the educators' resources, enabling them to be conducted without a program or equipment wherever their work takes place. The dropout rates were low, indicating the possible meaningfulness of the intervention for the educators.

This study has limitations. The educators participated in the study voluntarily, which may increase the possibility of selection bias because the participants may have already been motivated and thus may not reflect the real study population. The study instruments consisted of developed items for this study, lacking further validity analysis. Furthermore, the conceptual issue, the resource-workload-balance, needs more investigation and further instrument development. There was only a 1-month follow-up period due to the educators' summer break and, consequently, has no results for the long-term effectiveness. Randomization of the participants was not possible, as the study needed specific organizations to be comparable and the educators in the IG and the CG to be in different schools. Although it was challenging to recruit the educators, the number of educators remained sufficient to demonstrate the effectiveness of the outcome of this intervention.

## Conclusion

The findings indicate that applying accessible exercises during working hours could benefit the OW of nurse educators. The need for further development of this intervention with a longer follow-up period and further inspection of the intervention's usability and utility is required for sustaining the stability of the intervention.
